# High‐Flow Oxygen Therapy to Support Inpatient Pulmonary Rehabilitation During Very Severe Hepatopulmonary Syndrome Recovery Post Liver Transplant: A Case Report

**DOI:** 10.1002/ccr3.70472

**Published:** 2025-04-21

**Authors:** Jack M. Reeves, Jessica Marouvo, Aveline Chan, Nicholas Thomas, Lissa M. Spencer

**Affiliations:** ^1^ Graduate School of Health, Faculty of Health University of Technology Sydney Sydney New South Wales Australia; ^2^ Physiotherapy Department Royal Prince Alfred Hospital Sydney New South Wales Australia; ^3^ Sydney School of Health Sciences, Faculty of Medicine and Health The University of Sydney Sydney New South Wales Australia

**Keywords:** case report, hepatopulmonary syndrome, intrapulmonary vascular dilatation, rehabilitation

## Abstract

This case study reports the novel use of inpatient pulmonary rehabilitation (PR) with near‐maximal high‐flow oxygen therapy in a patient recovering from very severe hepatopulmonary syndrome (HPS) following liver transplantation. HPS is a rare condition where advanced liver disease alters lung microvasculature through intrapulmonary vascular dilatation (IPVD) and angiogenesis. Platypnoea–orthodeoxia (postural dyspnoea with concurrent blood oxygen desaturation) is characteristic of HPS due to redirection of blood flow to the basal lung where IPVDs are more prominent, secondary to gravity. Currently, the only definitive treatment is liver transplantation, which allows normalization of oxygenation over an extended period, typically within 1 year. Pulmonary rehabilitation is an effective intervention for improving dyspnoea, health‐related quality of life (HRQoL), and exercise capacity in people with chronic respiratory disease. Despite this, little is known of the effect PR has on individuals recovering from HPS post liver transplant. The aim is to describe an inpatient PR program for a patient recovering from HPS. This case study describes a 27‐year‐old male with “very severe” HPS who undertook inpatient PR 5 months posttransplant. The patient completed an 8‐week program of twice‐weekly PR supported by high‐flow oxygen therapy (fraction of inspired oxygen of 90%). He performed aerobic and resistance exercises for the upper and lower limbs in recumbent, seated, and standing positions. The patient improved in exercise capacity on the 1‐min sit‐to‐stand test (+4 repetitions), lower limb strength on the 5‐repetition sit‐to‐stand test (−3.4 s) and in HRQoL outcomes assessed. Following rehabilitation, the patient still had a high burden of respiratory symptoms and required continuous high‐flow oxygen therapy. This case study demonstrates that inpatient PR, modified for HPS‐associated platypnoea–orthodeoxia and supported by high‐flow oxygen therapy, is safe and effective and therefore feasible for other HPS patients.

**Summary**

- This case demonstrates that inpatient PR supported by high‐flow oxygen therapy, is safe and effective for those recovering from HPS following liver transplantation.
- Whilst severe HPS patients may be incapable of continuous exercise due to significant platypnoea–orthodeoxia and rapid exertional desaturation, the use of intervals of activity over a protracted period may allow an adequate training stimulus.

## 1 Introduction

Hepatopulmonary syndrome (HPS) is a rare disorder caused liver dysfunction that affects lung vasculature. It is characterized by intrapulmonary vascular dilatation (IPVD) and abnormal angiogenesis, which result in hypoxemia through ventilation–perfusion mismatch, diffusion restriction, and arteriovenous shunts [1]. A complex interaction between the liver and lungs caused by advanced liver disease leads to IPVDs at the precapillary and capillary levels impairing blood oxygenation through the above mechanisms [2]. The IPVDs are more frequent in the basal lung and therefore gravitational redistribution of blood flow when upright further exacerbates HPS pathophysiology [3]. This results in profound platypnoea–orthodeoxia (postural dyspnoea with concurrent blood oxygen desaturation) in the absence of identifiable lung disease [4], which can be severely physically limiting for patients. Table 1 outlines pathophysiological mechanisms of HPS.

**TABLE 1:** Mechanisms of HPS pathophysiology.

Mechanism	Pathophysiology
Ventilation–perfusion mismatch	Increase in the radius of blood vessels via intrapulmonary vascular dilatation results in a reduction in the resistance within the vessels (as per Pouiselle's law) increasing perfusion but with standard ventilation throughout the lung. This is the most prominent in the basal lung where perfusion is already greatest. Upright postures further increase basal perfusion (secondary to gravitational redirection) worsening ventilation–perfusion mismatch.
Diffusion restriction	Intrapulmonary vascular dilatation creates an increase in the diffusion distance for oxygen moving between the alveoli and hemoglobin molecules of red blood cells. Additionally, increased blood flow due to vascular dilatation (Pouiselle's law) leads to a shorter transit time for diffusion of oxygen.
Arteriovenous shunts	Abnormal angiogenesis (new blood vessel formation), attributable to enzymes released by the damaged liver, creates bypasses of the alveoli resulting in mixed oxygenated and de‐oxygenated blood.

Liver transplantation can reverse HPS physiological changes, improve survival [5] and remains the only definitive treatment [6]. Following liver transplantation, nearly all surviving HPS patients experience complete normalization of oxygenation despite a variable recovery time [7]. Whilst poorer preoperative arterial oxygen pressure (PaO_2_) could be associated with an increased rate of improvement postoperatively [7], most patients have resolution of HPS within 1 year [8]. Pulmonary rehabilitation (PR) is an effective intervention for people with chronic respiratory disease and has been shown to decrease dyspnoea, improve health‐related quality of life (HRQoL), and increase exercise capacity [9]. Exercise prescribed through PR programs allows the reversal of skeletal muscle dysfunction resulting from inactivity and deconditioning in people with respiratory limitations [10].

To the best of the authors knowledge, there is only one report of rehabilitation (reported in the year 2000) in a patient recovering from HPS following liver transplant, which differed from the present case in terms of oxygen therapy, patient severity, and program delivery [11]. As such, the safety, feasibility, and effectiveness of inpatient PR in supporting the natural recovery of this population is unknown in a modern context with the availability of high‐flow oxygen therapy.

## 2 Case History/Examination

This case study describes a 27‐year‐old male diagnosed with autoimmune hepatitis (AIH) at age 15 who went on to develop primary sclerosing cholangitis (PSC), a portal vein thrombosis (PVT), and subsequent severe HPS. The patient also had a history of ulcerative colitis with occasional exacerbations requiring hospitalization and steroid‐induced osteoporosis. He had no other significant past medical history.

Prior to liver transplantation, it is common for transplant candidates to be admitted for preoperative workup. During this admission, the patient was in hypoxic respiratory failure with an alveolar‐arterial gradient qualifying for HPS (> 15 mmHg diagnostic for HPS in patients < 64 years) with a PaO_2_ of 48 mmHg on arterial blood gas analysis (pH 7.52, PaCO_2_ 26 mmHg, HCO_3_ 21 mmol/L, SpO_2_ 86%, room air). This classified the patient as “very severe” (< PaO_2_ 50 mmHg). Additionally, the patient was treated for a right‐sided hepatic hydrothorax which resolved prior to discharge. He went home with supplemental oxygen (4 L per minute via nasal prongs). The patient had a model for end‐stage liver disease (MELD) score of 12.7.

On the day of transplant surgery, 1 month following preoperative workup, the patient presented to hospital profoundly hypoxic (following transport to hospital on room air). He had recurrence of his hepatic hydrothorax and deranged arterial blood gases despite recommencing 4 L of supplemental oxygen (pH 7.48, PaO_2_ 62 mmHg, PaCO_2_ 21 mmHg, HCO_3_ 16, SpO_2_ 92%). A liver transplant was performed using a split‐liver from a deceased donor and the patient remained intubated in the intensive care unit (ICU) for 21h postoperatively. Following extubation, the patient was placed on high‐flow oxygen therapy using a nasal cannula with a fraction of inspired oxygen (FiO_2_) of 75% and a flow of 30 L.

Whilst in ICU, the patient's FiO_2_ was slowly weaned to maintain SpO_2_ ≥ 90%. He was transferred to the ward 44 days postoperatively. At this time, he required continuous high‐flow oxygen therapy at 45% FiO_2_ with 30 L of flow whilst supine in bed. For ambulation < 10 m, he required 15 L of oxygen delivered through a non‐rebreather mask (NRM) followed by a 5 min recovery for oxygen resaturation. Weaning of FiO_2_ whilst maintaining target SpO_2_ ≥ 90% continued and the patient mobilized short distances (< 20 m) 1–3 times weekly on a NRM delivering 15 L of oxygen, under physiotherapy supervision.

### 2.1 Inpatient Pulmonary Rehabilitation

The patient was assessed for inpatient PR 156 days (~5 months) post liver transplant. At this time, he required high‐flow oxygen therapy at 35% FiO_2_ with 30 L of flow when in a semi‐fowlers position, and 60% FiO_2_ with 30 L flow while seated. He was unable to walk more than 20 m without desaturating to nadir SpO_2_ 75% on 15 L via NRM. A seated recovery time of 3 min was required for re‐saturation. Following assessment, the patient underwent an 8‐week, twice‐weekly inpatient PR program. Rehabilitation took place in a ward‐based therapy room with rehabilitation steps, light dumbbells, a recumbent exercise bike, and a chair. Sessions were approximately 1 h in duration and were supported by high‐flow oxygen therapy (90% FiO_2_, flow 30 L) using the portable AIRVO 3 (Fisher and Paykel Healthcare).

Both aerobic and resistance exercises were performed in recumbent, seated, and standing positions. The patient was also instructed to complete an additional independent program of bed‐based recumbent exercises. These were completed on three non‐rehabilitation days using elastic therapy bands. The bed‐based recumbent exercises prescribed are reported in Appendix S1.

### 2.2 Rehabilitation Outcomes

The 1‐min sit‐to‐stand test (1‐minSTST) was used as a measure of exercise capacity. Despite the 6MWT being the gold standard measure of exercise capacity in field walking tests [12], 6 min of upright exercise would have been intolerable for the patient. Prolonged ambulation would have caused severe platypnea–orthodeoxia and excessive exertional desaturation, based on recent episodes of short‐duration ambulation with minimal exertion despite maximal oxygen therapy. However, 1‐minSTST results have good correlation with the 6MWT [13] and are a valid measure of exercise capacity [14]. The Borg‐Dyspnoea scale [15] was used following the 1‐minSTST to evaluate exertional dyspnoea.

Muscular strength was assessed in the lower limbs using the 5‐repetition sit‐to‐stand test (5xSTST) [16] and in the upper limbs using grip strength dynamometry [17]. Baseline functional disability secondary to dyspnoea was evaluated using the modified Medical Research Council Dyspnoea Scale (mMRC‐Dyspnoea) [18]. Anxiety and depression were assessed using the Hospital Anxiety and Depression Scale (HADS) [19], a 14‐item validated questionnaire [20]. Health‐related quality of life (HRQoL) was measured using the 5‐level EuroQol (EQ‐5D‐5L) [21] and disease‐specific HRQoL using the Chronic Respiratory Disease Questionnaire (CRQ) [22] and the St George Respiratory Questionnaire (SGRQ) [23]. Respiratory symptoms were evaluated using the Chronic Airways Assessment Test (CAAT) [24]. The patient underwent a reassessment at 4 weeks (following 8 supervised sessions) and a final assessment at 8 weeks (following 16 supervised sessions) which included all rehabilitation outcomes. All physical testing was performed on a NRM delivering 15 L of oxygen.

## 3 Conclusion and Results

**FIGURE 1:**
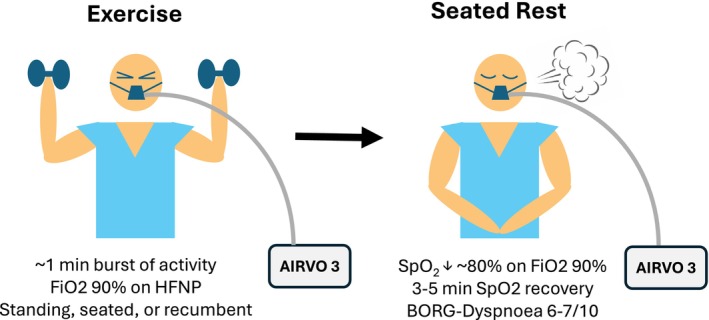
Cycle of exertional oxygen desaturation and recovery on near‐maximal high‐flow oxygen therapy. FiO_2_, fraction of inspired oxygen; HFNP, high‐flow nasal prongs; SpO_2_, blood oxygen saturation.

The patient successfully completed 16 sessions of PR over the course of 8 weeks with no adverse events. Exercises performed in each session of supervised rehabilitation are reported in Appendix S2. Exercises performed during rehabilitation were approximately 1 min in duration. The patient experienced oxygen desaturation to between 75% and 85% following each exercise (dependant on intensity and position). This was followed by a 3–5‐min rest period to allow for the target recovery of SpO_2_ ≥ 90% at FiO_2_ 90%, as seen in Figure 1. Rehabilitation outcomes at baseline, mid‐point, and completion of PR are shown in Table 2.

**TABLE 2:** Rehabilitation outcomes at assessment timepoints.

Outcomes	Baseline	4‐weeks	8‐weeks
1‐minSTST			
Pretest Borg‐Dyspnoea	0/10	0/10	0/10
Repetitions	20	22	24
Posttest Borg‐Dyspnoea	7/10	7/10	7/10
Nadir SpO_2_	70%	69%	69%
Time to 90% SpO_2_ recovery, mins	3:00	3:20	3:15
5xSTST			
Seconds	8.7	7.0	5.3
Grip strength (average of 3)			
Right (kg)	23.2	23.7	22.4
Left (kg)	21.5	23.8	27.5
mMRC‐dyspnoea	4/4	4/4	4/4
HADS			
Anxiety	0	1	0
Depression	6	6	4
CAAT	13	16	12
EQ‐5D‐5L			
Mobility	4	3	4
Personal care	3	3	3
Usual activities	4	4	4
Pain/discomfort	2	2	1
Anxiety/depression	1	1	1
Total health score	40	60	65
SGRQ			
Symptoms	46	15	13
Activity	93	74	80
Impact	35	58	42
Total	54	56	49
CRQ			
Dyspnoea	16	15	18
Fatigue	23	24	26
Emotion	41	42	43
Mastery	23	24	25

*Note:* All testing was performed on a non‐rebreather mask delivering 15 L of oxygen.

Abbreviations: 1‐minSTST, 1‐min sit‐to‐stand test; CAAT, Chronic Airways Assessment Test (previously COPD Assessment Test); EQ‐5D‐5L, EuroQoL, CRQ, Chronic Respiratory Questionnaire; HADS, Hospital Anxiety and Depression Scale; mMRC, modified Medical Research Council Dyspnoea Scale; SGRQ, St George Respiratory Questionnaire; SpO_2_, blood oxygen saturation; SPPB, Short Physical Performance Battery.

### 3.1 Physical Outcomes

The patient demonstrated improvement greater than the minimum clinically important difference (MCID) in exercise tolerance using the 1minSTS (4 repetitions, MCID 2.5 repetitions [25]) and in lower‐limb strength using the 5xSTST (−3.4 s, MCID 1.7 s [26]). Note the MCIDs [25, 26] reported are for PR populations who have chronic obstructive pulmonary disease, as there are no MCIDs reported for people with HPS. At all test points during the 1‐minSTST, the patient had a similar nadir oxygen desaturation (range: 69%–70%), posttest Borg‐Dyspnoea score (7/10 points), and oxygen recovery time to SpO_2_ 90% (range: 3:00–3:20 min). He also demonstrated a steady improvement in left grip strength but not right, with final assessment scores below age and sex matched normative values bilaterally [17].

### 3.2 Patient‐Reported Outcome Measures

The patients mMRC‐dyspnoea score was maximal at 4/4 at all testing timepoints. The patient was a non‐case for anxiety and depression on initial assessment using the HADS and remained a non‐case throughout, with slight changes seen between testing timepoints. The patient had a high burden of respiratory symptoms seen on the CAAT, which remained high on reassessment.

There was no pre‐post change on the EQ‐5D‐5L for the mobility, personal care, usual activities, and anxiety/depression domains; however, the pain/discomfort domain decreased by 1 point. There was a steady improvement in the EQ‐5D‐5L total health score from 40 to 65. The SGRQ demonstrated a pre‐post improvement in the “symptoms” domain and in the “activity” domain but not in the “impacts” domain. The total score of the SGRQ improved by 5 points. The CRQ scores for the domains of dyspnoea, fatigue, emotion, and mastery, all demonstrated moderate to large pre‐post improvement, based on the MCID [27].

## 4 Discussion

The patient demonstrated significant improvements in exercise tolerance (1‐minSTST) and lower limb strength (5xSTST), as well as significant improvements in HRQoL, as measured by the EQ‐5D‐5L total health domain, SGRQ, and CRQ. Despite this, the patient had a consistently high burden of respiratory symptoms (mMRC‐dyspnoea, CAAT).

Whilst orthodeoxia is characteristic of HPS, it is unknown whether recumbent or upright exercise is preferable for exercise‐related physiological adaptations. A protocol for a randomized controlled trial which is currently underway has hypothesized that HPS patients (of moderate or greater severity) will tolerate increased exercise time at a set workload, and therefore will achieve greater physiological adaptations, in recumbency compared to upright positions [28]. We utilized a program of mixed recumbent, seated, and standing (including ambulatory) exercise. This was because our patient was a young 27‐year‐old male who was several months post liver transplant with goals to return to normal activities. Additionally, he was mostly bed‐bound outside of sessions due to his “very severe” HPS, and was able to be adequately supported during exercise on maximal high‐flow oxygen therapy, and therefore was more engaged with a multipositional program.

Liver transplantation and time (in the absence of any exercise regime) have been shown to correct negative HPS‐related outcomes on cardiopulmonary exercise testing (CPET), such as decreased peak oxygen uptake, ventilation–perfusion mismatch, and hypoxemia [29]. However, given prolonged sedentarism, an inpatient PR program is likely to safely attenuate muscle strength and endurance decline, potentially augmenting ongoing physical recovery posthospital discharge. For this patient, resistance training during HPS recovery was also important given his background of steroid‐induced osteoporosis [30].

One other published case study described rehabilitation in a patient recovering from HPS post liver transplant, but with distinct differences from the present case [11]. Kohzuki et al. described a 17‐year‐old female who completed supine exercise that involved lifting the upper and lower limbs against gravity, diaphragmatic breathing techniques, and therapist‐assisted passive range of motion exercises (for contracture management). After 106 days, she was deemed to have made adequate recovery to begin an incremental exercise program without supplementary oxygen, but with the aim to maintain SpO_2_ > 85%. The program involved short‐distance ambulation, cycle ergometry for 10 min, and stair climbing. The Kohzuki case study reported a more conservative approach than the current case. They waited for refractory hypoxia to resolve prior to an incremental exercise program, whereas we utilized near‐maximal high‐flow oxygen therapy to facilitate an exercise program of greater intensity despite our patient's condition being significantly more severe at the time of participation. The patient in our case required 90% FiO_2_ when upright for approximately 1 min, and 35% FiO_2_ when resting supine in bed, to maintain SpO_2_ > 90%. Despite this, our patient was able to safely participate in a comprehensive program of rehabilitation in multiple positions due to the use of high‐flow oxygen therapy and structured work/rest periods based on exertional saturation. This means we were able to provide rehabilitation earlier in the patient's recovery, which for this patient was a protracted recovery due to their HPS severity.

A strength of our case study is the detailed rehabilitation information provided in a patient with “very severe” HPS, which can help guide clinicians in prescribing rehabilitation for people with HPS and refractory hypoxia post liver transplant. A limitation of this case study was the lack of CPET to evaluate physiological exercise constraints. However, physical testing in severe or very severe HPS is difficult as patients are often incapable of sustaining work for long durations, especially whilst upright, leading to difficulties in measuring physical outcomes. We used short (≤ 1 min) sit‐to‐stand tests allowing us to measure exercise capacity and lower limb strength with tolerable, yet near maximal, exertional dyspnoea and blood oxygen desaturation.

It was only recently that the first case report to demonstrate successful use of high‐flow oxygen therapy in a patient with HPS and severe posttransplant hypoxemia was published [31]. The current case report describes not only the use of high‐flow oxygen therapy in such a patient, but its successful use in facilitating participation in an inpatient PR program without adverse events. Despite platypnoea–orthodeoxia and rapid exertional desaturations, the patient in this case was able to achieve a therapeutic exercise workload. We achieved this by using short intervals of activity followed by adequate rest, supported by near‐maximal high‐flow oxygen therapy, regardless of the type or position of exercise employed.

### 4.1 Lessons Learned

Severe HPS patients can tolerate multi‐positional exercise when supported by high‐flow oxygen therapy, as the patient has in our case. When undertaking PR, these patients are likely to require long rehabilitation sessions due to exertional oxygen desaturation and associated time for recovery, a challenge when rehabilitation is delivered in an inpatient setting.

This case study demonstrated the safe, effective, and tolerable use of inpatient PR supported by near‐maximal high‐flow oxygen therapy and modified for HPS‐associated platypnoea–orthodeoxia in a patient with very severe HPS post liver transplantation. The use of PR to counteract sedentary‐related physical decline is feasible and should be considered in HPS patients recovering post liver transplantation.

## Author Contributions

**Jack M. Reeves:** conceptualization, data curation, formal analysis, investigation, methodology, project administration, writing – original draft, writing – review and editing. **Jessica Marouvo:** conceptualization, data curation, investigation, methodology, project administration, writing – review and editing. **Aveline Chan:** conceptualization, data curation, investigation, methodology, project administration, writing – review and editing. **Nicholas Thomas:** conceptualization, data curation, investigation, methodology, project administration, writing – review and editing. **Lissa M. Spencer:** conceptualization, data curation, investigation, methodology, project administration, supervision, writing – review and editing.

## Consent

We have obtained written patient consent for the publication of this case study.

## Conflicts of Interest

The authors declare no conflicts of interest.

## Supporting information


Appendix S1.

Appendix S2.

## Data Availability

The authors have nothing to report.
