# Supplementary material for: High‐Flow Oxygen Therapy to Support Inpatient Pulmonary Rehabilitation During Very Severe Hepatopulmonary Syndrome Recovery Post Liver Transplant: A Case Report
Source: Clin Case Rep. 2025 Apr 21;13(4):e70472. doi: 10.1002/ccr3.70472 (PMC12012242; doi:10.1002/ccr3.70472)
Supplement: Supplementary file 1 — Appendix S1. [file CCR3-13-e70472-s002.docx]

## Supplementary Document 1

## Recumbent Exercise Program

***
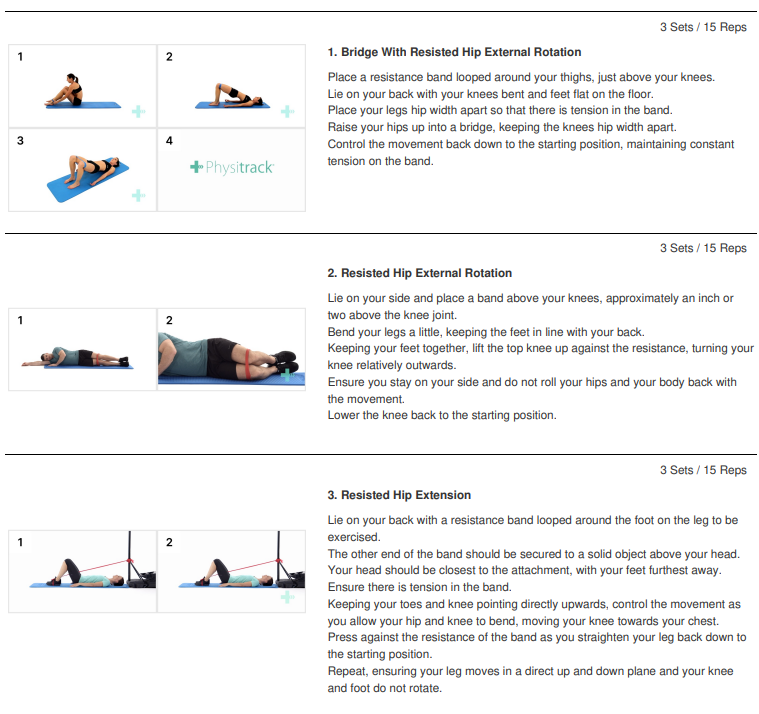
****Note: this program was completed three times per week, on non-gym days. All exercises were completed in a recumbent position*.

**
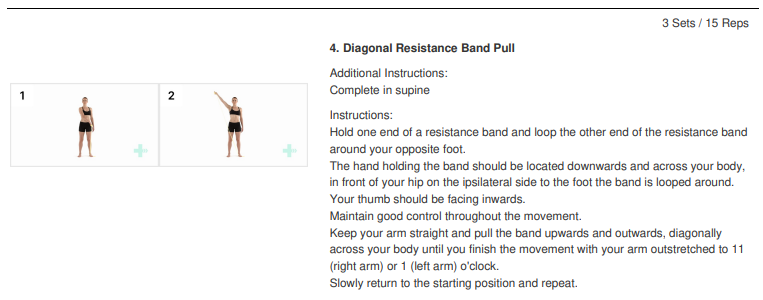
**

**
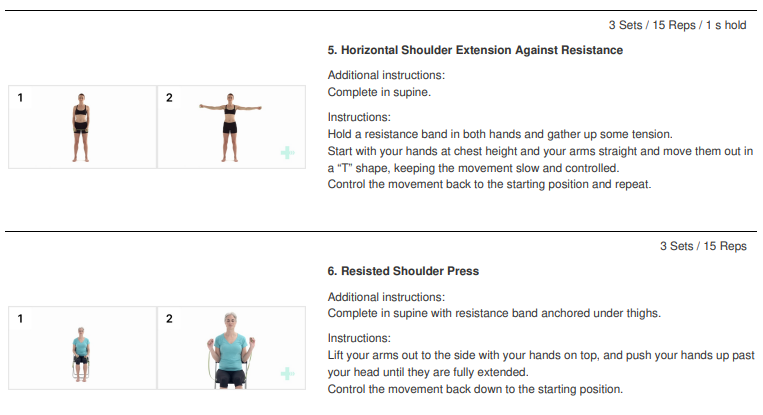
**
