# Supplementary material for: High‐Flow Oxygen Therapy to Support Inpatient Pulmonary Rehabilitation During Very Severe Hepatopulmonary Syndrome Recovery Post Liver Transplant: A Case Report
Source: Clin Case Rep. 2025 Apr 21;13(4):e70472. doi: 10.1002/ccr3.70472 (PMC12012242; doi:10.1002/ccr3.70472)
Supplement: Supplementary file 2 — Appendix S2. [file CCR3-13-e70472-s001.docx]

| **Week** | **Session**  **No.** | **Exercises Performed** | **Session**  **No.** | **Exercises Performed** |
| --- | --- | --- | --- | --- |
| 1 | 1 | 1. 3x sets of 10x reps bicep curls in standing 2kg  2. 3x sets of 12x step-ups  3. 3x sets of 10x reps shoulder press in standing 2kg  4. 3x sets of 10x reps sit-to-stand + ball catch and throw | 2 | 1. 2x sets of 50m ambulation  2. 2x sets of 10x step ups with high knee  3. 2x sets of 12x reps standing bent over row (green TheraBand)  4. 2x sets of 12x reps standing banded pull-apart (green TheraBand) |
| 2 | 3 | 1. 3x sets of 10x reps bicep curls in standing 3.5kg  2. 3x sets of 12x step-ups  3. 3x sets of 10x reps shoulder press in standing 3.5kg  4. 3x sets of 10x reps sit-to-stand + ball catch and throw  5. 3x sets of 1minute recumbent cycling (resistance level 4) | 4 | 1. 3x sets of 5x reps squats with 2kg overhead push press  2. 3x sets of 6x reps incline push-ups (45 degrees)  3. 3x sets of 12x step-ups  4. 3x sets of 10x reps sit-to-stand + ball catch and throw  5. 2x sets of 50m ambulation |
| 3 | 5 | 1. 3x sets of 10x reps bicep curls in standing 3.5kg  2. 3x sets of 12x step-ups  3. 3x sets of 6x reps incline push-ups (45 degrees)  4. 3x sets of 10x reps sit-to-stand + 3.5kg shoulder press  5. 3x sets of 1minute recumbent cycling (resistance level 4) | 6 | 1. 1x sets of 50m ambulation  2. 2x sets of 10x step ups with high knee  3. 2x sets of 12x reps standing bent over row (green TheraBand)  4. 2x sets of 12x reps standing pull-apart (green TheraBand) |
| 4 | 7 | 1. 2x sets of 10x reps standing overhead tricep ext 2kg  2. 2x sets of 10x reps dead lifts 2kg dumbbells  3. 2x sets of 10x reps standing bent over rows 2kg  4. 2x sets of 8x reps alternating lunges with 2kg dumbbells  5. 2x sets of standing balloon volleyball ~1 min | 8 | 1. 3x sets of 10x reps sit-to-stand + 3.5kg shoulder press  2. 3x sets of 6x reps incline push-ups (45 degrees)  3. 3x sets of 12x step-ups  4. 3x sets of 1minute recumbent cycling (resistance level 4)  5. 3x sets of 10x reps sit-to-stand + ball catch and throw |
| 5 | 9 | 1. 3x sets of 8x step-ups  2. 3x sets of 6x reps incline push-ups (45 degrees)  3. 3x sets of 10x reps seated lateral raise 3.5kg dumbbells  4. 3x sets of 10x reps bicep curls in standing 3.5kg  5. 3x sets of 1minute recumbent cycling (resistance level 4) | 10 | 1. 3x sets of 12x step-ups  2. 3x sets of 10 reps body weight squats  3. 3x sets of 6x reps incline push-ups (45 degrees)  4. 3x sets of 10x reps seated lateral raise 3.5kg dumbbells  5. 3x sets of 10x reps bicep curls in standing 3.5kg |
| 6 | 11 | 1. 2x sets of 8 reps alternating lunges with 2kg dumbbells  2. 3x sets of 10 reps body weight squats  3. 3x sets of 6x reps incline push-ups (45 degrees)  4. 3x sets of 10x reps seated lateral raise 3.5kg dumbbells  5. 3x sets of 10x reps bicep curls in standing 3.5kg | 12 | 1. 3x sets of 10x reps sit-to-stand + 3.5kg shoulder press  2. 3x sets of 15x step-ups  3. 3x sets of 10x reps sit-to-stand + ball catch and throw  4. 3x sets of 10x reps seated lateral raise 3.5kg dumbbells |
| 7 | 13 | 1. 3x sets of 10x reps sit-to-stand + 3.5kg shoulder press  2. 3x sets of 10x reps seated lateral raise 3.5kg dumbbells  3. 3x sets of 6x reps incline push-ups (45 degrees)  4. 3x sets of 12x step-ups  5. 3x sets of 10x reps sit-to-stand + ball catch and throw | 14 | 1. 3x sets of 10x reps sit-to-stand + 3.5kg shoulder press  2. 2x sets of 12x reps standing bent over row (blue TheraBand)  3. 2x sets of 12x step-ups holding 2kg  4. 3x sets of 6x reps incline push-ups (45 degrees)  5. 2x sets of 10x reps sit-to-stand + ball catch and throw |
| 8 | 15 | 1. 3x sets of 10x reps sit-to-stand + 3.5kg shoulder press  2. 2x sets of 12x step-ups holding 3.5kg  3. 3x sets of 10x reps seated lateral raise 3.5kg dumbbells  4. 3x sets of 6x reps incline push-ups (45 degrees)  5. 3x sets of 10x reps sit-to-stand + ball catch and throw | 16 | 1. 2x sets of 10x reps sit-to-stand + 3.5kg shoulder press  2. 2x sets of 12x reps bent over row 3.5kg  3. 2x sets of 12x reps alternating lunges with 3.5kg dumbbells  4. 2x sets of 10x reps incline push-ups (45 degrees)  5. 2x sets of 12x reps sit-to-stand + ball catch and throw |

Exercises were performed on high-flow oxygen therapy with fraction of inspired oxygen 90%.

The patient performed 3 additional sessions of independent exercise weekly that were separate to this log (See Supplementary Document 1).
